# Guiseppe Paglia, Guiseppe Astarita (Eds.): Ion mobility-mass spectrometry—methods and protocols

**DOI:** 10.1007/s00216-020-02479-x

**Published:** 2020-02-19

**Authors:** Oliver J. Schmitz

**Affiliations:** 1 Applied Analytical Chemistry, University of Duisburg-Essen, Universitaetsstr. 5, 45141 Essen, Germany; 2 Teaching and Research Center for Separation, University of Duisburg-Essen, Universitaetsstr. 5, 45141 Essen, Germany

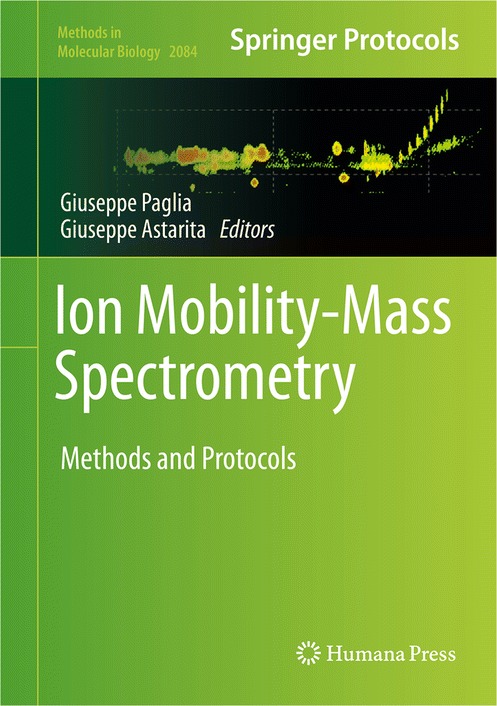



**Bibliography**

Ion mobility-mass spectrometry—methods and protocols

Series: Methods in Molecular Biology, Vol. 2084

Giuseppe Paglia, Giuseppe Astarita (Eds.)

Humana Press

ISBN 978-1-07-160029-0

Hardcover, 312 pages,

2020, €181.89

## Book’s topic

The triumphant progress of ion mobility-mass spectrometry (IM-MS) began about 15 years ago. In the meantime, more and more scientific studies show that the combination of ion mobility spectrometry with mass spectrometry has many advantages, e.g. in the field of non-target analysis. The advantages of the combination with chromatographic analysis methods are (i) the orthogonal separation between chromatography and ion mobility. This allows an extremely powerful two-dimensional separation to be achieved without the need for extensive method development. In addition, (ii) the determination of the collision cross section (CCS) helps to identify the analytes and (iii) IM-MS often enables the separation of isobaric substances (even without prior chromatographic separation). However, it is becoming increasingly apparent that the use of pre-separation also leads to a significant increase in performance of IM-MS. One- and two-dimensional gas and liquid chromatography and capillary electrophoresis have already been coupled with IM-MS. Of course, pre-separation can also be omitted and the sample inlet can be done via ambient ion sources such as DART and DESI. The book *Ion mobility-mass spectrometry—methods and protocols* by G. Paglia and F. Astarita was written by international experts in the field of IM-MS and gives an excellent overview of the status quo in bioanalysis with IM-MS.

## Contents

The 312-page book starts with an excellent introduction to the basics of IM-MS, followed by four sections with a total of 19 chapters. In the Introduction, various ion mobility spectrometers (DTIMS, TWIMS, and DMS) are described. Unfortunately, the relatively new TIMS could probably not be included here due to time constraints. In the first section, the use of IM-MS for metabolomics and lipidomics is discussed. In the 2nd and 4th chapters of this section, the DTIMS is discussed in particular detail. In the other chapters, the main focus was put on the methods and the protocol, and thus the lipid analysis by HILIC, the mycotoxin analysis by LC, and the analysis of cyclosporin A by direct injection with nano-ESI, each coupled with TWIMS- or DMS-MS, were described. The second section deals with the benefits of IM-MS in proteomics and glycomics and presents a short overview of the application area in this field. Thus, the application of IM-MS for the investigation of effects of protein modification and small molecule binding on protein dynamics is discussed in the same way as the in situ analysis of intact proteins by liquid extraction surface analysis with FAIMS. This section is rounded off with a chapter about ion mobility-mass spectrometry of glycoconjugates. The third section describes the coupling of IM-MS with ambient ion sources and the use of imaging IM-MS. In the 14th chapter, the desorption atmospheric pressure photoionization (DAPPI) is used as an ambient ion source. This ion source allows the ionization of medium to non-polar analytes. Analogous to this, the following chapter shows that laser ablation electrospray ionization (LAESI) can ionize rather polar analytes. An innovative feature is shown in the last chapter of this section, the use of MALDI with the TWIMS-MS, which significantly enhances the usual imaging due to the additional separation dimension. In the last part, bioinformatic solutions for analyzing IM-MS data and derived CCS values are shown. Here, the very useful LipidIMMS Analyzer is presented, which allows the CCS calculation of lipids. The next chapter describes the acquiring and interpretation of MS^3^ data for structure elucidation of metabolites. The book ends with a technical overview of the High-Performance Collision Cross Section software.

## Comparison with the existing literature

*Ion mobility spectrometry - mass spectrometry* by C. L. Wilkins and S. Trimpin from 2007 and *Advances in ion mobility – mass spectrometry: fundamentals, instrumentation and applications* from Q. A. Donald and J. S. Prell (2019) are two textbooks about the theory and instrumentation in this new and innovative field. The major difference of the book from G. Paglia and G. Astarita to these ones is the focus on methods and protocols, which allows the reader to start very fast in the own research by using the well-described protocols.

## Critical assessment

The book covers the most important areas of bioanalytics and is helpful for the newcomer to quickly and successfully apply the quite new ion mobility-mass spectrometry. For the expert user, however, it is also useful due to the different fields of application and the well-elaborated protocols. However, it does not replace a book on the theory of ion mobility-mass spectrometry, because only the DTIMS is described in more detail.

## Readership recommendation

As mentioned before, the book is very helpful to guide a newcomer quickly in the successful use of ion mobility-mass spectrometry. But there are also many useful tips about work flows in the field of proteomics, glycomic, and metabolomics/lipidomics including data analysis, which makes the book valuable even for more experienced users.

## Summary

The 19 chapters of this book provide detailed insights into the workflow of bioanalysis with ion mobility-mass spectrometry. The big advantages of this book are the detailed protocols which allow a very fast and successful start in a new analytical field. In addition, it is worth mentioning that each chapter in this book begins with a short introduction to the respective research area with many very useful references.

